# Inverted repeats in the monkeypox virus genome are hot spots for mutation

**DOI:** 10.1002/jmv.28322

**Published:** 2022-11-25

**Authors:** Michaela Dobrovolná, Václav Brázda, Emily F. Warner, Stefan Bidula

**Affiliations:** ^1^ Institute of Biophysics of the Czech Academy of Sciences Brno Czech Republic; ^2^ Faculty of Chemistry Brno University of Technology Brno Czech Republic; ^3^ School of Pharmacy, University of East Anglia Norwich Research Park Norwich UK

**Keywords:** APOBEC, evolution, inverted repeats, monkeypox, mutation

## Abstract

The current monkeypox virus (MPXV) strain differs from the strain arising in 2018 by 50+ single nucleotide polymorphisms (SNPs) and is mutating much faster than expected. The cytidine deaminase apolipoprotein B messenger RNA editing enzyme, catalytic subunit B (APOBEC3) was hypothesized to be driving this increased mutation. APOBEC has recently been identified to preferentially mutate cruciform DNA secondary structures formed by inverted repeats (IRs). IRs were recently identified as hot spots for mutation in severe acute respiratory syndrome coronavirus 2, and we aimed to identify whether IRs were also hot spots for mutation within MPXV genomes. We found that MPXV genomes were replete with IR sequences. Of the 50+ SNPs identified in the 2022 outbreak strain, 63.9% of these were found to have arisen within IR regions in the 2018 reference strain (MT903344.1). Notably, IR sequences found in the 2018 reference strain were significantly lost over time, with an average of 32.5% of these sequences being conserved in the 2022 MPXV genomes. This evidence was highly indicative that mutations were arising within IRs. This data provides further support to the hypothesis that APOBEC may be driving MPXV mutation and highlights the necessity for greater surveillance of IRs of MPXV genomes to detect new mutations.

## 1 INTRODUCTION

We are currently facing the first multi‐country outbreak of monkeypox and have much to uncover about this emerging threat. Since the start of the outbreak and as of November 12, 2022, 79 231 confirmed cases have been reported worldwide.^1^ This number continues to rise, and we must obtain a deeper understanding of what may be contributing to the evolution and propagation of the current monkeypox virus (MPXV) strain.

Isidro et al.,^2^ recently highlighted that the current 2022 MPXV strain was closely related to the MPXV strain exported from Nigeria to the UK, Israel, and Singapore in 2018/2019. Notably, they found that the 2022 strain differed from the 2018/2019 strain by around 50 single‐nucleotide polymorphisms (SNPs). As the reference sequence from 2018 only differs from the current sequence by approximately 100 bp, the mutation rate was between 6 and 12‐fold more than expected over this time period. These mutations were primarily G > A and C > T mutations, which they concluded was likely due to the activity of apolipoprotein B messenger mRNA (mRNA) editing enzyme, catalytic subunit B (APOBEC3) family members. APOBEC3 is a cytidine deaminase with innate antiviral activity that is upregulated during viral infections. This enzyme promotes G > A and C > T hypermutations at ‘hot spots' within viral DNA to render the virus less infective and prevent biological processes such as replication.^3^ However, there is also evidence that sublethal mutagenesis can contribute to greater genetic diversity and enhance viral propagation. Only a single mutation observed in the study above was not a G > A or C > T transition, highly indicative that APOBEC was involved in driving this mutational diversity.

There is growing evidence that non‐B DNA secondary structures such as cruciform (formed by inverted repeats [IRs]), triplexes, and G‐quadruplexes (G4) are involved in driving mutational diversity.^4^, ^5^, ^6^, ^7^ IRs are not to be confused with the inverted terminal repeats (ITRs), repeat sequences of around 2–12 Kbp which can occur within the first and last 12 Kbp of poxvirus genomes. In this instance, the terminal repeat at the 3′ end is complementary to the terminal repeat at the 5′ end of the entire genome sequence. In contrast, IRs are much shorter sequences and can be found interspersed throughout the entire genome. IRs consist of a single‐stranded sequence of nucleotides, followed downstream by its reverse complement, and separated by a short loop sequence consisting of any nucleotide (e.g., 5′‐AAGCTnnnnnAGCTT‐3′). When the loop length is zero, the sequence is referred to as a palindrome. IRs have been demonstrated to play important roles within genome instability, where they contribute to evolution and disease.^8^, ^9^, ^10^

Indeed, it was recently identified that mutations in severe acute respiratory syndrome coronavirus 2 (SARS‐CoV‐2) occurred with greater frequency within IRs and suggested that IRs are important drivers of viral mutational diversity.^11^ Interestingly, a recent study identified that APOBEC mutagenic activity was much higher against IRs compared with other non‐B or B‐DNA structures.^12^ Thus, one could question whether APOBEC might be driving mutational diversity in MPXV by inducing mutations within IRs. Here, we analyzed 247 MPXV genomes to identify the presence of both G4s and IRs. Furthermore, we identified which of the SNPs identified in the 2022 outbreak genomes arose within IR regions in the 2018 reference strain and whether IRs were hot spots for mutation in MPXV.

## 2 MATERIALS AND METHODS

### 2.1 Genome sequences

Two‐hundred and forty‐seven genomes were obtained from the National Center for Biotechnology Information (NCBI) database and analyzed for the presence of IRs and G4s (last accessed 11/11/2022; Table S1). One‐hundred and twenty‐four of these genomes were chosen at random to explore which IRs in the 2018 reference strain (MT903344.1) were conserved in MPXV genomes between 2018 and 2022. These genomes were representative of all currently known lineages; A (13 genomes), A.1 (13 genomes), A.1.1 (2 genomes), A.2 (3 genomes), A.3 (1 genome), B.1 (30 genomes), B.1.2 (6 genomes), and 5 genomes each for B.1.1, B.1.3‐B.1.12.

### 2.2 Detection of G4‐forming sequences in MPXV genomes

Analysis of genomes for G4‐forming sequences was conducted using G4Hunter.^13^ G4Hunter identifies all sequences with propensity to form G4 within a genome. The number of G4‐forming sequences present was identified at the detection thresholds 0–1.2, 1.2–1.4, 1.4–1.6, 1.6–1.8, 1.8–2, and above 2. The window size was 25 nucleotides. Those appearing at higher thresholds had a higher propensity to fold into G4s and those with a near‐zero average score were indicative of sequences likely to form duplexes. This data can be found in Table S2. To identify the location of G4‐forming sequences within annotated genomic features, the files containing known genomic features in the MPXV genomes were downloaded from the NCBI database. The presence of G4‐forming sequences within a pre‐defined genomic feature (e.g., gene), or within ±100 bp of these genomic features were analyzed. The location of G4‐forming sequences in known genomic features was identified using a publicly available script found at https://pypi.org/project/dna-analyser-ibp/.

### 2.3 Detection of IR sequences in MPXV genomes

Genomes were analyzed using Palindrome Analyzer to detect the presence and localization of IRs.^14^ The default parameters for analysis were to detect IRs with a size between 6 and 30 bp, spacer size from 0 to 10 bp, and with up to one mismatch. Information about the number and frequency of IRs within the MPXV genomes can be found in Table S3. The information regarding the nucleotide position of the SNPs in the 2018 reference strain was obtained from Isidro et al. and was cross‐referenced with our IR analyses to identify whether these were located within an IR sequence. Whether these exact IR sequences were conserved amongst other MPXV genomes between 2018 and 2022 was further manually assessed.

### 2.4 Statistics

Data were first tested for normality via a Shapiro–Wilk normality test. To assess whether IRs were being lost compared with the 2018 reference strain (MT903344.1), all data were normalized to this strain (mean of 100%) and significance was determined via a one‐sample *t*‐test. A *p*‐value of <0.05 was considered statistically significant.

## 3 RESULTS

**Figure 1:**
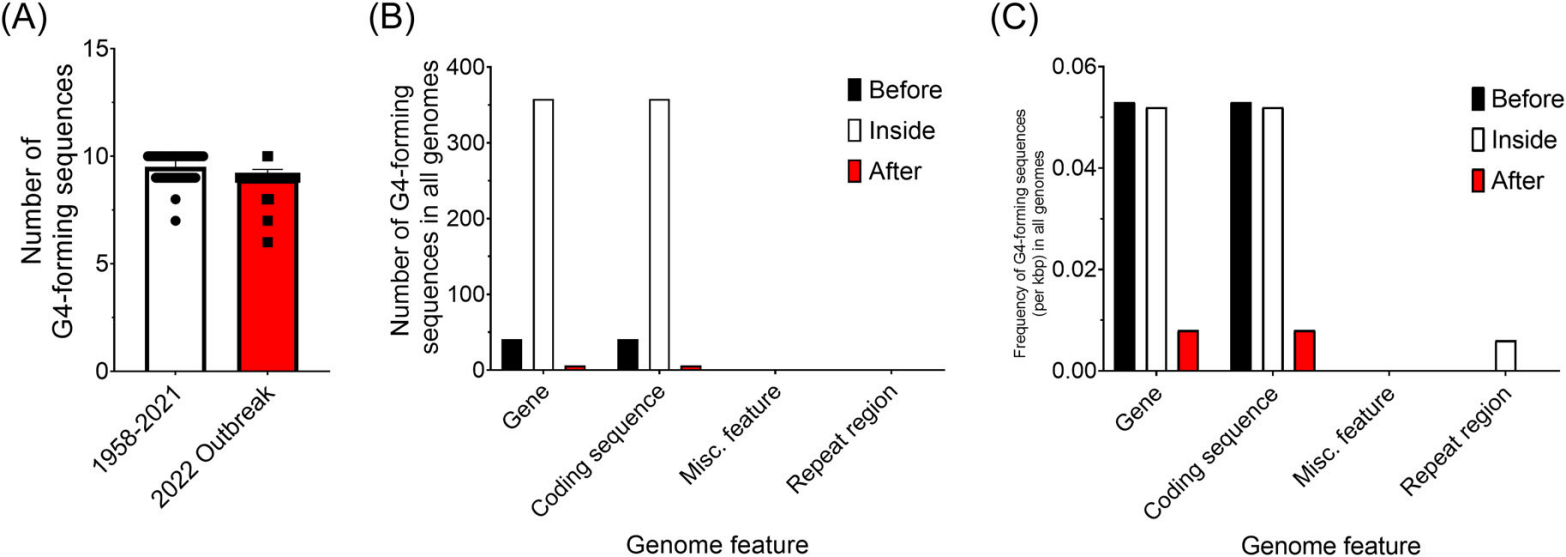
The number and location of predicted G4‐forming sequences in monkeypox virus (MPXV) genomes. (A) A comparison between the predicted number of G4‐forming sequences in MPXV genomes obtained between 1958 and 2021 and the current 2022 outbreak. Individual points represent a single MPXV genome, and the columns represent the mean. The number (B) and (C) frequency of G4‐forming sequences per kbp located within annotated genomic features. Data in (B) and (C) represent the combined total of all genomes analyzed. Error bars represent the SD. Data is representative of 136 individual genomes collected between 1958 and 2022.

It has previously been reported that members of the *Poxviridae* family have some of the lowest frequencies of G4‐forming sequences among viruses.^15^ However, it has also recently been shown that all MPXV genomes from the 2022 outbreak contain an unstable G4 in the C9L gene, which increases inhibition of the immune response.^16^ We first analyzed MPXV genome sequences for the presence of G4‐forming sequences. As expected, we identified very few G4‐forming sequences within these genomes, ranging from 6 to 10 sequences (frequency of 0.030–0.055 per kbp; Figure 1A; Table S2). Most of these sequences were found within 100 bp before genes and within genes themselves, with very few sequences being identified in the 100 bp following the gene sequence (Figure 1B,C).

**Figure 2:**
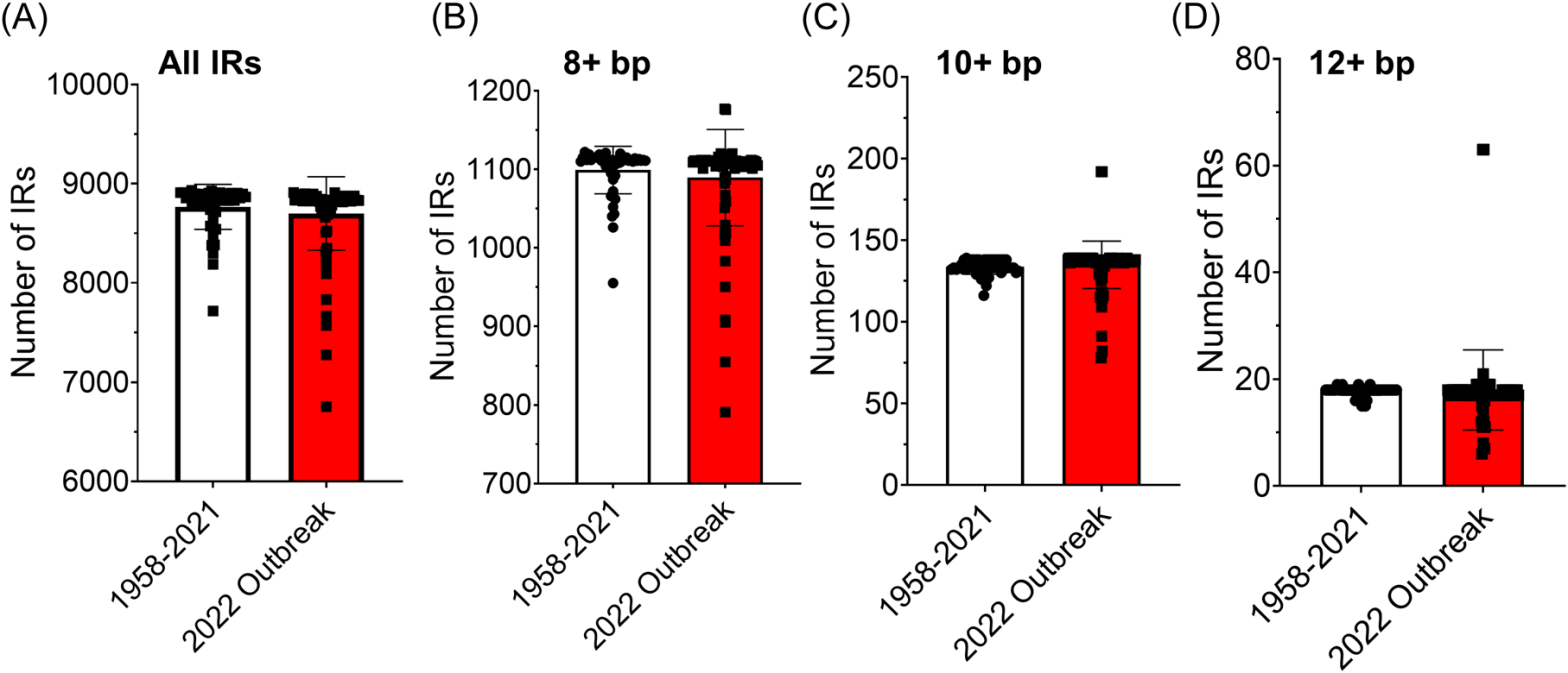
The number of inverted repeat (IR) sequences in monkeypox virus (MPXV) genomes. (A) A comparison between the total number of IR sequences in MPXV genomes obtained between 1958 and 2021 and the current 2022 outbreak. Individual points represent a single MPXV genome. (B–D) The number of IRs composed of 8+, 10+, or 12+ nucleotides. Error bars represent the SD. Data are representative of 136 individual genomes collected between 1958 and 2022.

Next, we identified the number of IR sequences. Unlike G4‐forming sequences, MPXV genomes were found to be replete with IRs and the number of IRs within MPXV genomes ranged from 6754 to 8933 (frequency of 34.25–45.11 per kbp; Figure 2A; Table S3). Although not significantly different, we observed that there was a small increase in the number of MPXV genomes from 2022 with fewer IRs of all sizes compared to the older genomes (Figure 2B–D). Peculiarly, there were two genomes (ON609725.2 and ON631241.1) that had far greater numbers of these longer IRs (63 IRs of 12+ bp compared to an average of 18 amongst other genomes). Both these MPXV samples were obtained from Slovenia.

The 2022 outbreak MXPV strains are thought to be closely related to the 2018 strain exported from Nigeria to the UK, Israel, and Singapore. The 2022 outbreak strains differ from this strain by 50+ SNPs which are likely to have originally appeared in the 2018 strain between 2018 and 2022. We next assessed whether the 50+ SNPs found in the 2022 outbreak MPXV strains were found to arise inside (or within 3 bp) of an IR sequence found in the 2018 reference strain genome (MT903344.1). We found that 63.9% of the SNPs were found to arise within IRs (Table 1). Of these, 100% of the intergenic, 71.9% of the nonsynonymous, 45.5% of the synonymous, and 0% of the stop‐gained mutations fell within these IR sequences.

**Table 1:** SNPs from the 2022 strain arising within IR sequences found in the 2018 reference genome (MT903344.1)

Type of mutation	Nucleotide position	Inverted repeat region?	Sequence (Stem‐Loop‐Stem)
Nonsynonymous	1271	NO	
Nonsynonymous	2600	YES	**CTGGAAGCGTAAGTTCCCG**
Synonymous	3120	NO	
Synonymous	3531	YES	**TCGTATCCGATGTATACGA**
Nonsynonymous	3827	YES	**ACAATCAAGATTAT**
Synonymous	7780	YES	**ATCGACGGTATGTATTGTAGAT**
Nonsynonymous	14009	CLOSE	**CTATTAACCATTCTATTAG**
Intergenic	15437	YES	**CTATAGAATCAAAACACGATAG**
Synonymous	16977	NO	
Nonsynonymous	18952	YES	**GTTGATTATTTCTGACATCGAC**
Synonymous	21732	NO	
Synonymous	30376	NO	
Nonsynonymous	31062	YES	**TTTGGCGTAAATGTGTGCGAAA**
Nonsynonymous	34468	YES	**GAAGTAATGAAATCACTTC**
Synonymous	37211	YES	**TATAACTGAACTGAGATATA**
Synonymous	38369	NO	
Nonsynonymous	38671	YES	**CAATACCGTATCG**
Nonsynonymous	39148	NO	
Synonymous	52894	YES	**ATCTGACTAAGAT**
Nonsynonymous	54126	CLOSE	**AAATTCATCCATGGTGGCATTT**
Nonsynonymous	54644	YES	**GACAATAATGTC**
Nonsynonymous	55084	YES	**ACATACATCGTCGGTATTT**
Nonsynonymous	55142	YES	**ATATTAACGAGTTCCATTTATAT**
Synonymous	64306	YES	**ATCGATTTTCAAATCCAT**
Synonymous	64435	YES	**ACGCGTCGTTAACTCGT**
Nonsynonymous	73075	CLOSE	**GTCTATAAATGTAGAC**
Nonsynonymous	73248	YES	**TGCTATCATAGATATAGAA**
Nonsynonymous	74214	YES	**ATACGTTCGATATGAACATAT**
Nonsynonymous	77392	NO	
Synonymous	81284	NO	
Synonymous	82383	YES	**TCAAAATGCTGATTTCGA**
Synonymous	82460	NO	
Synonymous	82862	NO	
Synonymous	84596	NO	
Nonsynonymous	89915	YES	**TGAAGAAAATTCTCCA**
Nonsynonymous	94807	YES	**TATTTTTTTCTGAATATA**
Synonymous	95043	CLOSE	**CCATCATTAGGAGATGATAG**
Nonsynonymous	124139	YES	**GTCTAGTATTCGAGAC**
Nonsynonymous	124683	NO	
Stop gained	127229	NO	
Nonsynonymous	128707	NO	
Intergenic	144538	CLOSE	**TTATTATAAAATAA**
Nonsynonymous	150480	YES	**GATGAATTTGATC**
Intergenic	151472	YES	**TATTATTTTCAGTTTTATTATA**
Intergenic	155806	CLOSE	**ATAATTTTATAGATTAT**
Synonymous	162342	NO	
Nonsynonymous	167528	YES	**ATGTACCAGAAGGAACAT**
Synonymous	170273	NO	
Nonsynonymous	176910	NO	
Intergenic	178220	YES	**AATGATATACGTAACATT**
Synonymous	181382	YES	**CAGAAATTATCTCTG**
Nonsynonymous	181995	NO	
Nonsynonymous	183534	NO	
Nonsynonymous	186593	CLOSE	**ATGACTATCTTGAATCAT**
Intergenic	187169	YES	**TTAGAATACTTTCCGAATAAGTCTTCTAA**
Nonsynonymous	190675	YES	**TGAAGAATTTTTCA**
Nonsynonymous	193407	YES	**TGATTGTACACCCATCA**
Synonymous	193703	YES	**TCGTATACATCGGATACGA**
Synonymous	194114	NO	
Nonsynonymous	194634	YES	**CGGGAACTTACGCTTCCAG**
Nonsynonymous	195953	NO	

Abbreviations: IRs, inverted repeats; SNPs, single nucleotide polymorphisms.

**Figure 3:**
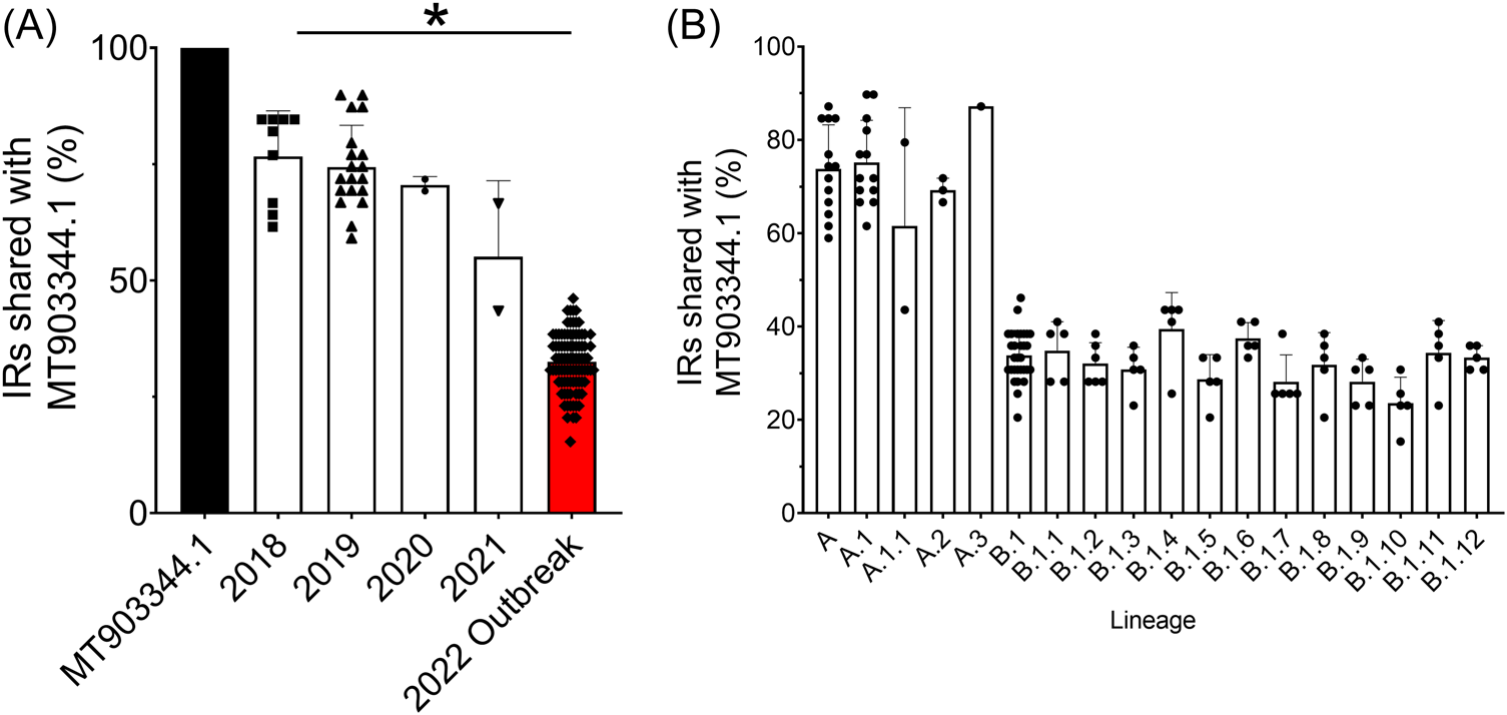
IRs are hot spots for monkeypox virus (MPXV) mutations. (A) MPXV genomes obtained in 2018, 2019, 2020, 2021, and 2022 were analyzed to determine whether the inverted repeats (IR) sequences in the 2018 reference genome (MT903344.1) where single nucleotide polymorphisms (SNPs) arise were conserved. The percentage of conserved sequences was shown to decrease between 2018 and 2022, indicating that these IR sequences were mutating over time. Data are representative of 124 genomes collected in 2018 (10 genomes), 2019 (19 genomes), 2020 (2 genomes), 2021 (2 genomes), and 2022 (91 genomes). (B) The percentage of conserved IR sequences listed by genetic lineage. Error bars represent the SD. Each point represents an individual genome. Normality was tested via a Shapiro–Wilk normality test and significance was determined via one‐sample *t*‐test. *p* > 0.05 was considered statistically significant.

We then proceeded to identify whether the IRs observed in the 2018 reference strain were conserved amongst other MPXV genomes obtained in 2018 (9 genomes) and those from 2019 (19 genomes), 2020 (2 genomes), 2021 (2 genomes), and 2022 (91 genomes). Here, we investigated whether the exact IR sequence could be found, with the rationale that the loss of these exact sequences would be indicative of mutations occurring within these regions and altering the sequence. Surprisingly, we found that there was a time‐dependent loss of these sequences between 2018 and 2022 (Figure 3). The average conservation of these IR sequences in the 2018 strains was 76.6%, which decreased to 74.4% in 2019, 70.5% in 2020, 55.1% in 2021, and 32.5% in the 2022 strains (Figure 3). This was strongly indicative that mutations were arising in IRs over time and IRs were a hot spot for mutation in the MPXV.

However, not all IRs were lost/sites of mutation in the 2022 outbreak strains and some IRs were frequently conserved amongst strains. These included the IRs found within the Kelch domain protein (D18L), DNA polymerase (F8L), telomere‐binding protein (I6L), late transcription factor 4 (H5R), the virion core (E3R), a pseudogene, two within the surface glycoprotein (B21R), and three out of four of the intergenic mutations (Table S4).

## 4 DISCUSSION

In this study, we identified that MPXV genomes were depleted of G4s but replete with IR sequences. Additionally, we showed that almost two‐thirds of the new mutations observed within the 2022 outbreak strain originally arose within IRs in the 2018 strain and that IRs are hot spots of genetic variability in MPXV genomes. This data highlights that future mutations may further arise within IRs and that these regions should be monitored. Moreover, this new evidence further supports the hypothesis that APOBEC is driving mutations within MPXV genomes.

Previous observations have identified that the genomes of viruses within *Poxviridae* contain extremely low frequencies of G4‐forming sequences.^15^ However, the fact that the few which remain are conserved and influence biological functions is indicative of their importance within MPXV genomes. Yicong et al.,^16^ reported that the 2022 strains all contain an unstable variant of a G4 in C9L that causes an increase in C9L protein levels and likely increased inhibition of host immunity. Consequently, this would result in propagation of the virus. Based upon this evidence, compounds which stabilize the G4 secondary structure may have therapeutic value in inhibiting the protein expression of C9L and augmenting the innate immune response.

Conversely, MPXV genomes were replete with IRs. This observation agrees with our previous analysis of mitochondrial genomes, whereby the number of IRs in the genome were inversely proportional to the number of G4‐forming sequences.^17^ However, we believe the large number of long IR sequences occurring in the two genomes from Slovenia (ON609725 and ON631241) occurred as an artifact of the sequencing process. Both these genomes were sequenced simultaneously, came from two different lineages, and this observation was not made in other genomes from these lineages.

Notably, almost two‐thirds of the SNPs in the 2022 strain arose in an IR sequence in the 2018 reference strain. It is interesting to note that some of the IR sequences were more frequently retained amongst the 2022 outbreak strains. The exact reasons underlying this are unknown. Though it may be because mutations within these regions have not yet occurred, that some of these IRs are not hot spots of mutation but provide some structural importance, or that mutations to these regions would be detrimental to MPXV and they have evolved some mechanisms to try and prevent mutations at these sites within these genes.

APOBEC has high mutational activity against IRs and the exact IR sequences where the SNPs arise are lost over time. This is strong evidence that APOBEC‐IR interactions are likely critical in driving mutational diversity in MPXV. Previous evidence has suggested that APOBEC3G, F, and H have no activity against the vaccinia virus, with a primary focus on APOBEC3G.^18^ However, only 5 of 47 mutations identified by Isidro et al., were characteristic of APOBEC3G activity (GG > AG), with the other 42 being characteristic of the activity of other APOBEC3 family members (G > A).^2^ Thus, this suggests the influence of other APOBEC3 members (APOBEC3A‐D) could be important and the role of APOBEC3G is negligible. Unfortunately, experimental evidence describing the activity of these other APOBEC3 members is currently lacking and further investigation is necessary. However, one could hypothesize that unlike APOBEC3G, F, and H, there will be other APOBEC family members with activity against MPXV.

The primary role of APOBEC is to enhance antiviral immunity by inducing deleterious mutations, however, the evidence suggests that mutations within the MPXV genome may be driving propagation.^3^ As aforementioned, it has previously been observed that the induction of sublethal mutations within HIV‐1 can enhance its ability to adapt and propagate.^19^ Therefore, it may be that APOBEC is inducing mutations within the MPXV genome that are augmenting viral propagation, rather than preventing it. If this is true, then inhibitors of APOBEC could be a potential strategy to reduce the speed at which the MPXV genome is evolving.

Taken together, we provide convincing evidence that IRs are a predominant site of mutations within the MPXV genome. Furthermore, we provide support to the hypothesis that APOBEC is driving MPXV mutational diversity, potentially via its interaction with IRs in the mutation hot spots. This study highlights the importance of monitoring the formation of mutations within IR sequences and provides a potential link between APOBEC3 activity and mutation in MPXV genomes.

## AUTHOR CONTRIBUTIONS

Stefan Bidula, Emily F. Warner, and Václav Brázda developed the research question and analysis plan. Stefan Bidula, Václav Brázda, and Michaela Dobrovolná were involved in data collection and analysis. All authors were involved in preparing the final manuscript.

## CONFLICTS OF INTEREST

The authors declare that there are no conflicts of interest.

## Supporting information

Supplementary information.

Supplementary information.

Supplementary information.

Supplementary information.

## Data Availability

The data that support the findings of this study are available from the corresponding author upon request.
